# Recent developments in chemical diversity

**DOI:** 10.3762/bjoc.8.92

**Published:** 2012-06-06

**Authors:** John A Porco

**Affiliations:** 1 Boston University, Department of Chemistry, 590 Commonwealth Avenue, Boston MA 02215, USA

**Keywords:** chemical diversity

Diversity-oriented synthesis (DOS) is an important field involving the synthesis of libraries of diverse small molecules for applications including biological screening. The last decade has witnessed major progress in the field, with continued emphasis on structural and functional diversity for DOS library construction. A number of recent reviews have updated the community on this topic, including recent successes of biologically active molecules obtained from DOS compound collections [1–2].

This Thematic Series of the *Beilstein Journal of Organic Chemistry* attempts to capture recent developments in the area of chemical diversity and highlights the development of chemical reaction methodologies, the construction of novel chemotypes, and the advances in technology that enable progress in these areas. In the original paradigm for DOS, compound and library design were substantially based on the incorporation of functional, skeletal, and stereochemical diversity elements. An emerging theme in the area, as evident from many of the participating contributions in the Thematic Series, involves the development of novel reaction methodologies as a means to access or discover new “chemotypes”. In this way, organic chemists are empowered by the paradigm of “chemical diversity as a function of novel chemical reactions”.

“*Recent developments in chemical diversity*” in the *Beilstein Journal of Organic Chemistry* represents contributions from leading organic chemists exploring a diverse repertoire of approaches to access novel chemical diversity. I would like to thank all the authors for their important contributions to this issue and this vibrant area of research.

John A. Porco Jr.

Boston, May 2012
